# American Dental Convention

**Published:** 1862-10

**Authors:** 


### Editorial.

### AMERICAN DENTAL CONVENTION.

THE annual meeting of this body was held at Trenton Falls on the 5th, 6th, 7th and 8th of August, and it was a good meeting; if not better, it was certainly equal to any of the previous meetings, at least those we have attended. This is true, whether we consider the amount and character of the work accomplished, or the professional and social enjoyment of the members. Every one seemed just in his element, and determined that every body else should be.

The scenery of that locality is magnificently grand, and quite sufficient to repay a visit to it. In other respects, there was not much that was prepossessing. The accommodation, perhaps owing to the great number present—that's the scape-goat for all such sins—was not what it should have been, but then what was deficient in this respect was made *up* in the bills at the end! We do think the place was a poor one for the meeting. The friends of Trenton Falls who voted to take the Convention there last year were nowhere to be found; not one. We could not find a man who voted for it, or that knew a man who did, so we concluded Trenton Falls had no friends; in the Convention at least. But notwithstanding this, the meeting was a good one. The papers and discussions were very instructive and interesting. These meetings can always be good and valuable if the members will give their attention to the work.

Well,—that war meeting! It was *slightually* mixed. Matters and things generally were discussed. The extremes were handled without gloves; those who got farthest away from the subject for consideration did the tallest jumping—perhaps hoping to get back to it, or at least to see it.

The Jews, from father Abraham down, came in for a fair share of consideration. The man on the Jews, though less vehement than some others, did ample justice to his subject. The teeth and dentistry generally were duly considered; showing most conclusively that what is most in a man's mind will turn up on all occasions. But in the end, the right thing was done by a veteran who knew what to talk about, and how to say it.

Well, all things considered, the dental war meeting was a decided success, for it covered *all* the ground.

The next meeting of the Convention will be at Saratoga, a good place for a meeting, we know by experience, and we hope to meet a large number of our professional brothers there, and in as good condition as they were at Trenton Falls. T.

